# Potential association between membranous nephropathy and sargramostim therapy for pulmonary alveolar proteinosis 

**DOI:** 10.5414/CNCS108420

**Published:** 2014-12-15

**Authors:** Kamal Sewaralthahab, Helmut Rennke, Sarah Sewaralthahab, Nicolaos E. Madias, Bertrand L. Jaber

**Affiliations:** 1 Department of Medicine, Division of Nephrology, St. Elizabeth’s Medical Center,; 2 Department of Medicine, Tufts University School of Medicine,; 3 Kidney Pathology Service, Department of Pathology, Brigham and Women’s Hospital, Boston, MA, and; 4 Department of Internal Medicine, Division of Gastroenterology and Hepatology, Johns Hopkins University, Baltimore, MD, USA

**Keywords:** membranous nephropathy, pulmonary alveolar proteinosis, sargramostim, granulocyte-macrophage colony-stimulating factor

## Abstract

We present the case of a 43-year-old woman with a diagnosis of pulmonary alveolar proteinosis, on chronic treatment with sargramostim, a recombinant granulocyte-macrophage colony-stimulating factor, who presented with the nephrotic syndrome secondary to biopsy-proven membranous nephropathy. We discuss potential underlying mechanisms, including speculated effects of sargramostim on mesangial cells and the kidney resident macrophages, and review the existing literature on the potential association between these two disorders.

## Introduction 

Pulmonary alveolar proteinosis (PAP) is a rare respiratory disease, first described in 1958 [1], with ~ 700 cases reported as of 2010 [2, 3, 4]. It is characterized by the intra-alveolar accumulation of phospholipid and protein-surfactant material with minimal inflammation or fibrosis [1]. Congenital and acquired (idiopathic and secondary) forms of PAP exist, with idiopathic being the most common type. Impairment of surfactant clearance by alveolar macrophages resulting from inhibition of the granulocyte-macrophage colony-stimulating factor (GM-CSF) by blocking autoantibodies underlies many of the idiopathic cases, ultimately causing dysfunction of alveolar macrophages. The clinical course of PAP ranges from spontaneous resolution to progressive respiratory failure and death. Whole-lung lavage and GM-CSF administration are the main treatments of idiopathic PAP. 

We present the case of a 43-year-old woman with idiopathic PAP on chronic treatment with sargramostim (Leukine^®^, Sanofi-Aventis U.S LLC, Bridgewater, NJ, USA), a recombinant GM-CSF, who presented with the nephrotic syndrome, secondary to biopsy-proven membranous nephropathy, which went into full remission as the dose of sargramostim was tapered off. We discuss potential underlying mechanisms and review the existing literature on the association between these two disorders. 

## Case presentation 

A 43-year-old woman with idiopathic PAP confirmed by video-assisted thoracoscopic lung biopsy 2 years earlier had received two sessions of whole-lung lavage with minimal improvement. She was then commenced on daily injections of sargramostim (dose titrated up to 750 µg/d) with full resolution of her respiratory symptoms, discontinuation of home oxygen therapy, and normalization of pulmonary function tests by the 8^th^ month. At the 17^th^ month of continuous treatment, she presented with a 2-week history of dyspnea, lower-extremity swelling, weight gain, and nocturia. She was normotensive and had lower-extremity edema. The urinalysis revealed 4+ protein and fatty casts. Serum creatinine was normal (0.74 mg/dL) but she had low serum albumin (1.9 g/dL), elevated urine albumin-to-creatinine ratio (3,120 mg/g), and low density lipoprotein (LDL) cholesterol (173 mg/dL). 

**Figure 1.**
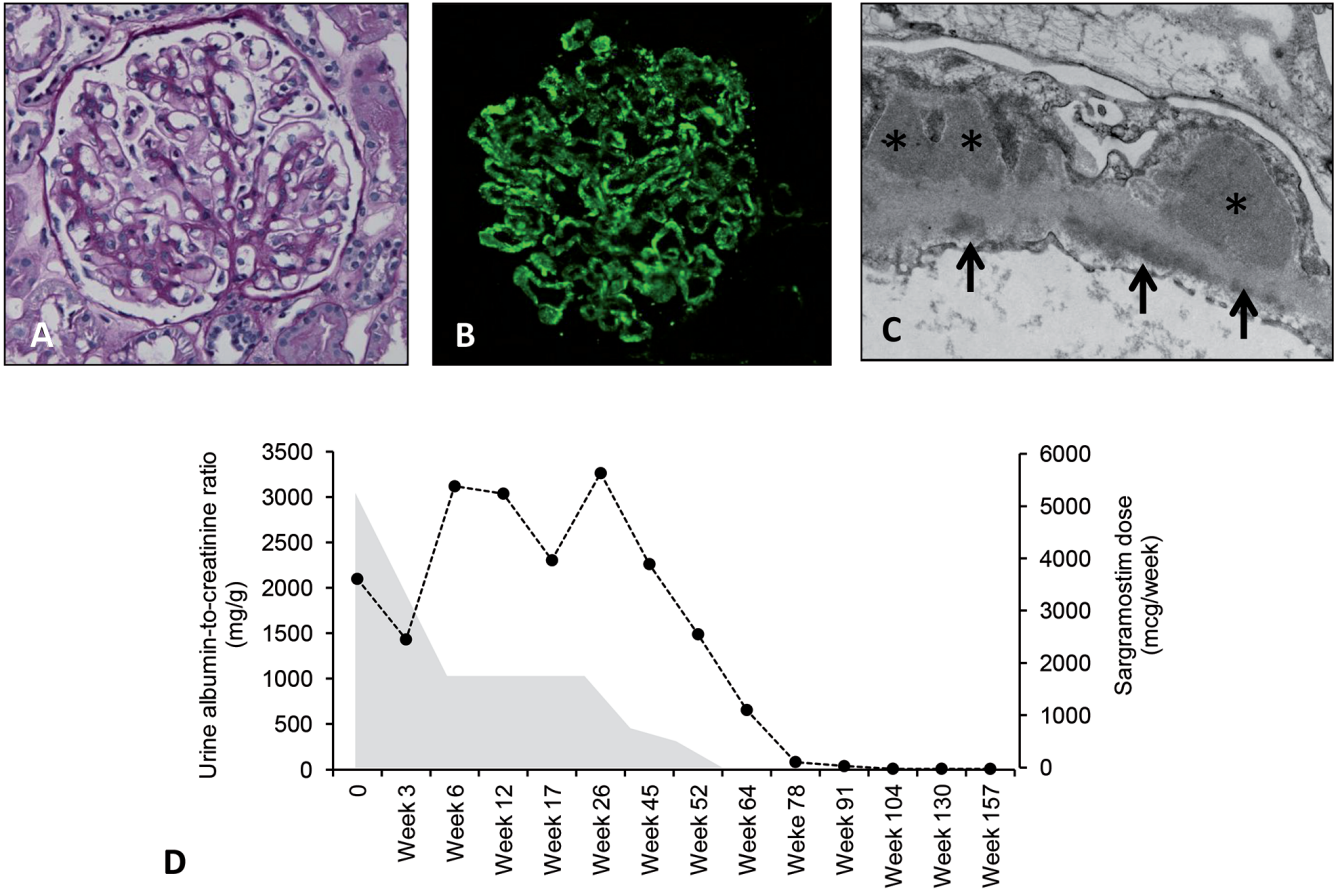
Kidney biopsy findings of membranous nephropathy and time course of disease. A: Light microscopy reveals glomeruli that are enlarged, with normal cellularity, and without signs of inflammation, fibrinoid necrosis, or sclerosis. The peripheral capillary walls reveal thickened basement membranes with spike-like projections on the silver-methenamine stain (not illustrated). There is no evidence of significant interstitial inflammation or fibrosis (PAS stain). B: Direct immunofluorescence microscopy reveals diffuse fine-granular deposition of IgG (illustrated) and less intense C3 and C1q staining (not illustrated) predominantly along the peripheral capillary walls. There is no reactivity for the PLA2R in these deposits (not illustrated). C: The electron micrograph shows a greatly distorted capillary wall, with numerous subepithelial electron-dense deposits (asterisks). Individual deposits are sometimes separated from each other by short basement membrane “spikes”. The electron dense deposits are finely granular, but they do not show organized substructures. There is extensive effacement of the visceral epithelial cell foot processes. There are also several subendothelial deposits present (arrows). D: Time course of proteinuria in relation to the tapering of the sargramostim dose. Hatched line indicates urine albumin-to-creatinine ratio (g/g), and shaded area indicates the weekly dose of sargramostim.

Kidney biopsy revealed an immune complex-mediated glomerulopathy, with a diffuse membranous pattern of injury, characterized by numerous subepithelial electron-dense deposits, frequent subendothelial and mesangial electron-dense deposits, and strong reactivity of the deposits for IgG, C3, and C1q, most suggestive of a secondary membranous nephropathy (Figure 1A-C). The serological work-up was unrevealing, including a negative screen for antinuclear antibody (ANA), hepatitis B and C virus infection, and normal complement levels. Circulating antiphospholipase A2 receptor (PLA2R) antibodies were absent, and no anti-PLA2R antibody renal deposits were detected by immunohistochemistry, suggestive of a secondary form of membranous nephropathy. The patient was not initiated on immunosuppressive therapy as she was normotensive, her kidney function was normal, and the proteinuria was < 4 g/day. Instead, she was treated conservatively with lisinopril, furosemide, simvastatin, and aspirin. After titrating the dose of lisinopril to 40-mg daily, the furosemide was stopped, and spironolactone 25-mg daily was added for antiproteinuric effect. In addition, considering that the membranous nephropathy might have been triggered not by PAP itself but by its treatment, the dose of sargramostim was progressively tapered off in close collaboration with the pulmonologist over the course of 1 year, which coincided with the full remission of the nephrotic syndrome (Figure 1D) and no clinical relapse of the PAP. Her medical regimen was de-escalated with the discontinuation of simvastatin and spironolactone and decrease of the lisinopril dose (10-mg daily). At the 3-year mark, her random urine albumin-to-creatinine ratio was 8 mg/g, and her PAP remained clinically inactive. 

## Discussion 

Membranous nephropathy is an autoimmune disease characterized by thickening of the glomerular capillary wall as a result of subepithelial deposits of immune complexes. Clinically, the disease is associated with nephrotic-range proteinuria and variable long-term clinical outcomes, ranging from spontaneous resolution of the proteinuria (observed in 1/3 of cases) to end-stage kidney failure [5, 6]. While the etiology of the disease is unknown and termed idiopathic in 75% of patients, the rest of the cases are termed secondary and have a recognizable etiology [7]. In 70% of patients with idiopathic membranous nephropathy, circulating autoantibodies are directed against PLA2R, which is localized to the podocyte surface, while the remaining 30% are deemed idiopathic with negative anti-PLA2R antibodies [8]. Our patient likely had secondary membranous nephropathy in light of the additional presence of subendothelial and mesangial deposits on electron microscopy and the absence of both circulating anti-PLA2R antibodies and anti-PLA2R-antibody renal deposits. 

A rare syndrome characterized by the accumulation of lipoproteinaceous material, mainly surfactant, within the alveoli, PAP results in impaired gas exchange and progressive respiratory failure. The accumulation of surfactant lipids is mainly due to macrophage dysfunction. The clinical course of the disease varies widely, from spontaneous resolution to death as a result of pneumonia or respiratory failure [9]. It is classified as congenital and acquired (idiopathic and secondary) [10, 11]. Congenital PAP is a heterogeneous group of hereditary disorders caused by mutations in the gene encoding surfactant proteins, the ABCA3 (ATP-binding cassette, sub-family A, member 3) transporter, and GM-CSF receptor [2, 12, 13, 14, 15]. Idiopathic PAP represents 90% of cases and is associated with circulating GM-CSF-neutralizing antibodies. Secondary forms occur in the setting of hematological malignancies, inhalation of toxic dust, fumes, or gases, immunosuppression due to infections or drugs, such as sirolimus [16, 17], and lysinuric protein intolerance, an autosomal recessive disease caused by defective transport of cationic amino acids [18]. These disorders can impair alveolar macrophage function, leading to surfactant accumulation [9]. The diagnosis of PAP involves a bronchoscopy with bronchoalveolar lavage showing large, foamy macrophages in Periodic Acid-Schiff (PAS)-positive fluid. Lung biopsy, which is the gold standard, shows intact parenchyma with macrophages present within alveoli filled with PAS-positive eosinophilic material staining positively for surfactant protein [11]. Sequential whole-lung lavage to mechanically remove the sediment is considered first-line therapy; it is effective in > 60% of cases and provides temporary symptomatic relief. However, it is associated with potential morbidity and fails to correct the underlying defect [19, 20]. In cases of secondary PAP, addressing the underlying cause is necessary. In 1996, the first case describing the efficacy of GM-CSF therapy for idiopathic PAP was reported [21], and a subsequent single-arm uncontrolled trial of 14 patients confirmed its efficacy [22]. Additional cases describing the efficacy of this therapy have been published [23, 24]. It is presumed that administration of GM-CSF promotes the proliferation and function of alveolar macrophages [24], which accelerate the clearance of surfactant lipids and proteins [25, 26]. 

Our review of the literature identified 6 reported cases of glomerulopathies associated with PAP (Table 1). These include 3 children with lysinuric protein intolerance-associated PAP who had the unexpected finding of immune complex-mediated glomerulonephritis at autopsy [18]. They were classified as diffuse glomerulonephritis, membranous nephropathy, and mesangioproliferative glomerulonephritis [18]. The remaining cases involved a young adult who developed secondary amyloidosis in the setting of longstanding PAP, succumbing to his illness, [27] and 2 cases of membranous nephropathy [28, 29]. The first of these cases was a 38-year-old woman who presented with a pulmonary-renal syndrome comprising diffuse bilateral pulmonary infiltrates and the nephrotic syndrome, with biopsy-proven diagnoses of PAP and membranous nephropathy, and found to have an immunodeficiency syndrome, characterized by the absence of monocytes in the peripheral blood and bone marrow [28]. Bone marrow-derived cells cultured ex vivo using GM-CSF failed to grow macrophage colonies, and as a result GM-CSF therapy was not offered. The patient eventually succumbed to her illness. The second case involved a 47-year-old woman presenting with nephrotic-range proteinuria, severe cough and fever, with evidence of bilateral pulmonary nodules and ground-glass opacities, and biopsy-proven diagnoses of PAP and membranous nephropathy [29]. While serum anti-GM-CSF antibodies could not be detected, the patient had circulating antibodies against alpha enolase, a cytoplasmic glycolytic enzyme expressed on the surface of monocytes and macrophages [30]. The patient refused treatment and was followed closely. Over the course of one year, she had a spontaneous remission of her nephrotic syndrome, the radiographic pulmonary findings improved, and the anti-alpha-enolase antibodies became undetectable. 

At the time of diagnosis of membranous nephropathy, both patients summarized above had active lung disease and were not receiving active therapy. In our case, at the time of diagnosis, the patient’s PAP was in clinical remission on sargramostim maintenance therapy. We suspect that sargramostim, rather than PAP, triggered development of membranous nephropathy. While Micromedex lists a rise in blood urea nitrogen level as a potential adverse effect of sargramostim, there are no published reports of kidney-related toxicity. However, a query in MedWatch, the U.S. Food and Drug Administration’s Safety Information and Adverse Event Reporting Program (www.FDAble.com), identified 2 reported cases – a 63-year-old man with membranoproliferative glomerulonephritis, and a 47-year-old man with membranous nephropathy – where sargramostim was the primary suspect. More details on these cases are unavailable. While the association between membranous nephropathy and PAP or its treatment might be coincidental, we speculate that in our case, sargramostim triggered development of membranous nephropathy, through direct or indirect effects on mesangial cells, which are the resident macrophages in the kidneys. The presence of mesangial immune deposits and the complete resolution of the proteinuria following tapering off of the drug are in support of this hypothesis. However, the possibility that the patient suffered from idiopathic membranous nephropathy, which underwent spontaneous remission over the course of 2 years, coinciding with the prolonged tapering period of sargramostim therapy, cannot be excluded. 

The potential role of GM-CSF in the pathogenesis of glomerular diseases is not well defined. Produced by monocytes, mesangial cells, and podocytes, GM-CSF enhances the proliferation and differentiation of progenitor cells and activates leukocytes at inflammatory sites, inhibiting their out migration and modulating cellular responses by stimulating the production of proinflammatory cytokines [31, 32, 33]. In studies of kidney biopsies obtained from patients with various forms of glomerulonephritis, increased GM-CSF tissue expression has been shown to correlate with glomerular proliferation, macrophage infiltration, and level of proteinuria [34]. In our patient, we can only speculate whether the exogenous administration of GM-CSF triggered or contributed to the development of an immune-complex-mediated glomerular injury. 

In conclusion, this case provides a possible link between sargramostim therapy for PAP and development of membranous nephropathy. This preliminary observation requires further study. 

## Conflict of interest 

There were no potential conflicts of interest noted. 

**Table 1.** Summary of the clinical, laboratory, and pathology features of 7 patients (including the current case report) presenting with glomerulopathies associated with pulmonary alveolar proteinosis.

Reference	Age/Sex	Type of acquired PAP	Relevant serological studies	Treatment of PAP	Serum creatinine (mg/dL)	Urinary protein finding	Kidney pathology finding	Specific treatment	Outcome
Ganguli et al. [27] (1972)	21/M	Idiopathic	–	None	–	2+ protein	Amyloidosis*	Prednisone	Death
Parto et al. [18] (1994)	10/F	Secondary (lysinuric protein intolerance	–	None	–	2.2 g/L	Diffuse Glomerulonephritis*	None	Death
	13/F	Secondary (lysinuric protein intolerance	–	None	0.85	–	Mesangial Glomerulonephritis*	None	Death
	7/F	Secondary (lysinuric protein intolerance	–	None	3.15	–	Membranous nephropathy*	None	Death
Witzke et al. [28] (2004)	38/F	Secondary (immunodeficiency syndrome with absence of monocytes)	–	Whole-lung lavage	Normal	4.2 g/day	Membranous nephropathy	Prednisone	Death
Yamada et al. [29] (2007)	47/F	Secondary	Absence of anti-GM-CSF antibody; presence of anti-α enolase antibody	None	0.56	3.2 g/day	Membranous nephropathy	None	Spontaneous remission of PAP and nephrotic syndrome
Sewaralthahab (present case) (2014)	43/F	Idiopathic	Absence of anti-PLA2R antibody	Whole-lung lavage and sargramostim therapy with clinical remission	0.74	3.1 g/g of creatinine	Membranous nephropathy	De-escalation of sargramostim therapy	Remission of nephrotic syndrome

PAP = pulmonary alveolar proteinosis; M = male; F = female; GM-CSF = granulocyte-macrophage colony stimulating factor; Note: Conversion factor for units: SCr in mg/dL to mmol/L, 388.4. *by autopsy.
